# Comparative Evaluation of Eustachian Tube Changes in Oral Submucous Fibrosis Patients

**DOI:** 10.7759/cureus.39040

**Published:** 2023-05-15

**Authors:** Anupama Pottam, Lavanya Dharmana, Damera Ajit, B. Badari Ramakrishna, Rahul Marshal Vaddeswarapu, K V Lokesh

**Affiliations:** 1 Department of Oral Medicine and Radiology, Anil Neerukonda Institute of Dental Sciences, Visakhapatnam, IND; 2 Department of Dentistry, Andhra Medical College, Visakhapatnam, IND; 3 Department of Oral Medicine and Radiology, Gitam Dental College, Visakhapatnam, IND

**Keywords:** hearing loss, cbct, eustachian tube, osmf, potentially malignant disorder

## Abstract

*Aim and Objective:* Oral submucous fibrosis (OSMF) is a potentially premalignant disorder affecting the oral cavity and its adjacent structures. The present study was aimed at a comparative evaluation of eustachian tube (ET) changes in OSMF patients using audiometry and cone-beam computed tomography (CBCT).

*Materials and Methods:* A total of 40 patients who were clinically diagnosed with OSMF were taken for the study and were graded into clinical and functional staging. After grading, the patients were subjected to audiometry to evaluate their hearing deficit. Subsequently, the patients were subjected to CBCT analysis for the evaluation of the length and volume of the ET. The length of ET was measured in the axial sections of the full-face CBCT images taken at the level of the root tip of the upper first molar. The radiolucency from the nasopharyngeal opening to the maximum distance was considered. The volume of ET was measured using third-party software (ITK-SNAP) in the radiolucent area.

*Result:* The age group in which a higher number of OSMF cases were seen was between 41 and 50 years. There was mild to moderate hearing loss observed in the right and/or left ear, with little variation between right and left ear changes in audiometry. The CBCT analysis did not show a significant difference in the mean length of the eustachian tube when comparing OSMF cases with normal. However, as the disease worsened, the length on the right and left sides significantly decreased. Additionally, there was no statistically significant difference in the mean eustachian tube volume between disease cases and controls. According to the clinical subgrades, the overall volume decreased from lower grade to higher grade, but there was no discernible difference between the left and right ear. The volume on function sub-grading between the right and left ear, however, was significantly reduced. Thus, the length and volume of ET decreased as the disease severity increased, but the mild to moderate hearing loss found in different clinical and functional grades of OSMF cases was not statistically significant.

*Conclusion:* Therefore, from the present study, it can be concluded that all OSMF cases should be evaluated for hearing deficit, and imaging of the eustachian tube should be part of the OSMF assessment for morphological changes of the ET that may cause hearing deficit.

## Introduction

Oral submucous fibrosis (OSMF) was first described by Schwartz in 1952 among five East African women of Indian origin [1]. It is a disease that is primarily associated with the chewing of areca nut, an ingredient in betel quid and is prevalent in South Asian populations [2]. It is a potentially malignant and crippling disorder of the oral mucosa, causing significant morbidity in terms of retardation of oral function as tissues become rigid and mouth opening becomes difficult. Mortality occurs when it transforms into squamous cell carcinoma. The introduction of gutkha, a type of chewing tobacco containing areca nut, into the market has been associated with a steep increase in the frequency of OSMF [3]. Studies have suggested that dysplastic changes are seen in about 25% of biopsied OSMF cases, and the rate of transformation to malignancy varies from 3% to 19% [4]. The primary presenting features of OSMF include a burning sensation in the oral cavity and reduced mouth opening.

The fibrotic changes in this condition are associated with increased collagen synthesis and reduced degradation of collagen. The various components regulating the pathogenesis of OSMF include areca nut alkaloids, leading to increased proliferation of fibroblasts; stabilization of collagens by the tannins; upregulation of the enzyme lysyl oxidase and raised copper levels, increasing collagen synthesis and its cross linkage; upregulation of cyclo-oxygenases and fibrogenic cytokines, along with inhibition of collagen phagocytosis and increased inflammatory activity [5]. All these factors may lead to the formation of fibroelastic changes in the oral musculature, leading to stiffness and the inability to open the mouth [6]. OSMF can involve the various muscles of the oral cavity, including the muscles of the floor of the mouth, hard and soft palate, oropharynx, and nasopharynx, such as the tensor veli palatini, the levator veli palatine, the tensor tympani, and the salpingopharyngeus [7]. The involvement of the soft palate can lead to changes within the cartilaginous component of the eustachian tube, which is a part of the soft palatal musculature, and hence previous studies have reported a loss of hearing in cases of OSMF [7].

It was hypothesized that increased fibrosis could lead to hearing loss in these patients. The current study was undertaken to evaluate the changes in the eustachian tube (ET) in patients with different grades of oral submucous fibrosis. Any dysfunction of the eustachian tube was assessed by audiometry in these patients [8]. Furthermore, no studies have been done on the evaluation of ET using cone-beam computed tomography (CBCT). The advent of CBCT as an imaging modality in the head and neck region has provided a novel way for the assessment of the morphological and anatomical changes in OSMF patients.

Hence, this study was undertaken to assess the length and volume of the eustachian tube using CBCT imaging in patients with various grades of OSMF.

## Materials and methods

A total of 40 cases who were clinically diagnosed with OSMF of either sex were selected from the outpatient department of Oral Medicine and Radiology, GITAM Dental College and Hospital, Rushikonda, Visakhapatnam, for the study with the Institutional Review Board number: IEC/GDCH/2021/174.

All the cases were clinically graded as per the grading criteria of More C et al. [9] for clinical as well as functional staging. All the study subjects were simultaneously sent for audiometric and CBCT analysis of the eustachian tube. CBCT images of 20 age- and sex-matched healthy controls were procured from the archives of the database, who previously came for CBCT examination for other dental problems.

The following inclusion criteria were followed: patients who were diagnosed clinically and confirmed histopathologically as having OSMF; age range from 18 to 70 years. The following exclusion criteria were followed: patients who gave a prior history of treatment for OSMF; patients with any other ear abnormalities or ear, nose, and throat (ENT) diseases; and patients with any history of major systemic illnesses or having an immunocompromised status.

**Figure 1:**
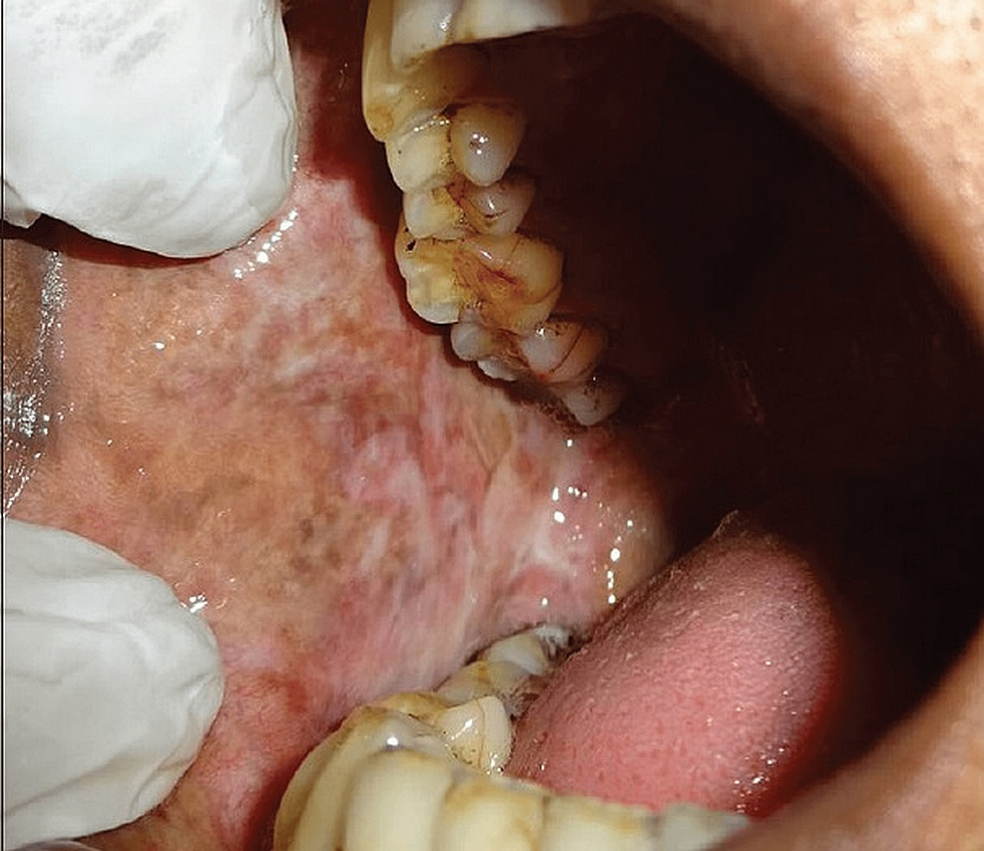
Oral submucous fibrosis clinical image

Procedure for clinical examination: All the patients selected for the study were made to sit comfortably on the dental chair. Conventional oral examinations were performed under incandescent operatory light using a mouth mirror, gauze, and digital palpation. All the cases were first examined under the guidance of senior faculty, diagnosed clinically as cases of oral submucous fibrosis, and then classified according to the classification given by More C et al. (2012) [9]. After obtaining an informed written consent form, the complete history was recorded in a prescribed format. A detailed clinical examination was done, and clinical staging was given as per More C et al. (2012) criteria as follows: Stage 1 (S1) - Stomatitis and/or blanching of the oral mucosa, Stage 2 (S2) - Presence of palpable fibrous bands in buccal mucosa and/or oropharynx, with/without stomatitis, Stage 3 (S3) - Presence of palpable fibrous bands in buccal mucosa and/or oropharynx and in any other parts of the oral cavity, with/without stomatitis, Stage 4 (S4) (a) - Any one of the above stages along with other potentially malignant disorders, e.g., oral leukoplakia, oral erythroplakia, etc., Stage 4 (S4) (b) - Any of the above stages along with oral carcinoma (Figure 1).

Then the maximum mouth opening was recorded by using a graduated scale and divider, measuring the distance between the upper and lower incisal edges (IID) in mm, and these patients were functionally staged as per More C et al. (2012) [9] criteria as follows: M1 - Interincisal mouth opening up to or > 35 mm, M2 - Interincisal mouth opening between 25 mm and 35 mm, M3 - Interincisal mouth opening between 15 mm and 25 mm, M4 - Interincisal mouth opening < 15 mm.

**Figure 2:**
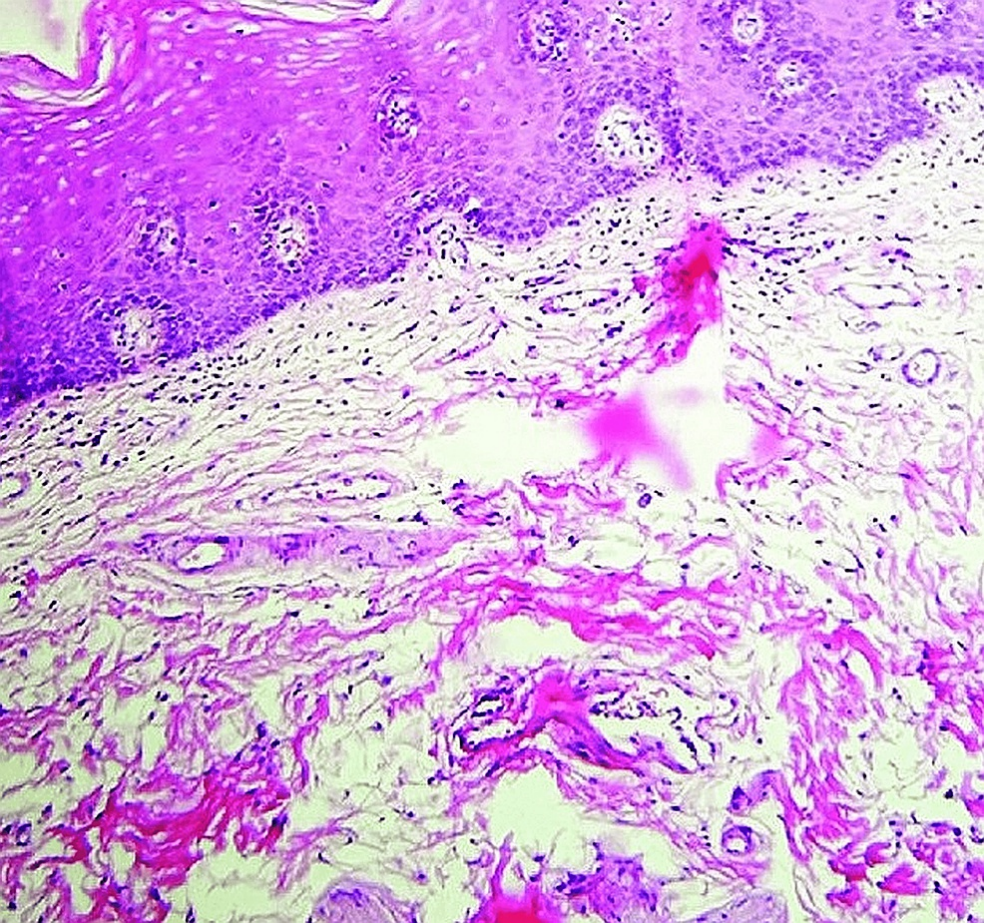
Histopathological changes in the oral submucous fibrosis patient

All 40 OSMF cases selected for the study that fulfilled the criteria of inclusion and exclusion were clinically staged as S1, S2, S3, and S4 and functionally staged as M1, M2, M3, and M4. All the pre-investigations were carried out before the punch biopsy, and the procedure of the biopsy was carried out under aseptic conditions. The tissue was sent for histopathological examination at the Department of Oral Pathology, GITAM Dental College and Hospital (Figure 2).

The patients were then sent to the ENT department at GITAM Institute of Medical Sciences & Research, Visakhapatnam, for a thorough examination of the ear by senior faculty of the department and were found to be normal on clinical examination. Then the patients were subjected to audiometric analysis for functional assessment.

**Figure 3:**
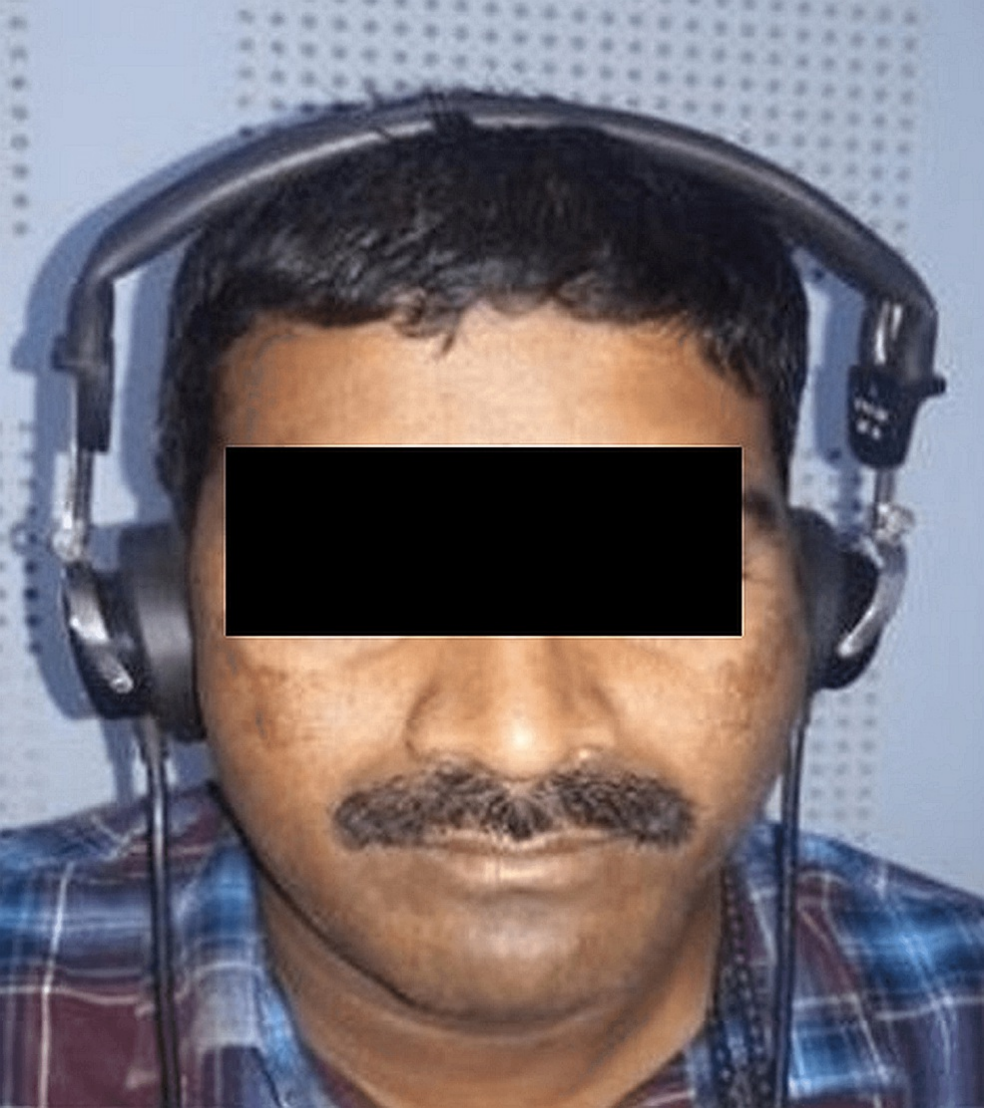
Pure tone audiometry machine and patient positioning

Procedure for audiometry: After taking a detailed clinical history and examination of the ears, the audiometry test was carried out. The patient was seated in a totally soundproof room, following which audio cups or earphone enclosure devices were placed over the ears for partial attenuation of environmental noise. The diaphragm of the headphones was placed exactly over the opening of the external auditory meatus. The ear in which the bone conduction test was being done was invariably left uncovered by the earphones. The bone conduction vibrator was attached to a spring metal headband and placed over the mastoid bone. The tension of the spring was such that the pressure over the mastoid by the headband was approximately 500mg per square centimeter. The area where the vibrator was placed should be as free of hair as possible. The patient was first familiarized with the tone by introducing the sound at the arbitrarily presumed suprathreshold level. If the patient hears the tone, then the tone is reduced in steps of 10 dB till the patient stops hearing or fails to give a positive response. Once this stage was reached, the tone was raised by 5 dB. If the patient heard this tone, then it was again decreased by 10 dB. If he does not hear it, the tone was again raised by 5 dB. In this way, by crossing several thresholds, the exact hearing threshold was obtained when one got at least three out of five responses correct. The second ear was tested similarly, starting with the last frequency used to test the first ear. The values of the threshold were plotted against the frequencies, and the graph was obtained for both ears separately. The red-colored line on the graph represents the right ear, and the blue-colored line on the graph represents the left ear. Once audiometry was completed, all the OSMF cases were subjected to CBCT (CS-9300, Care Stream Dental CBCT unit, Voxel size 180 μm, Kv 90, mA6.3, sec 8) analysis for eustachian tube evaluation (Figure 3).

**Figure 4:**
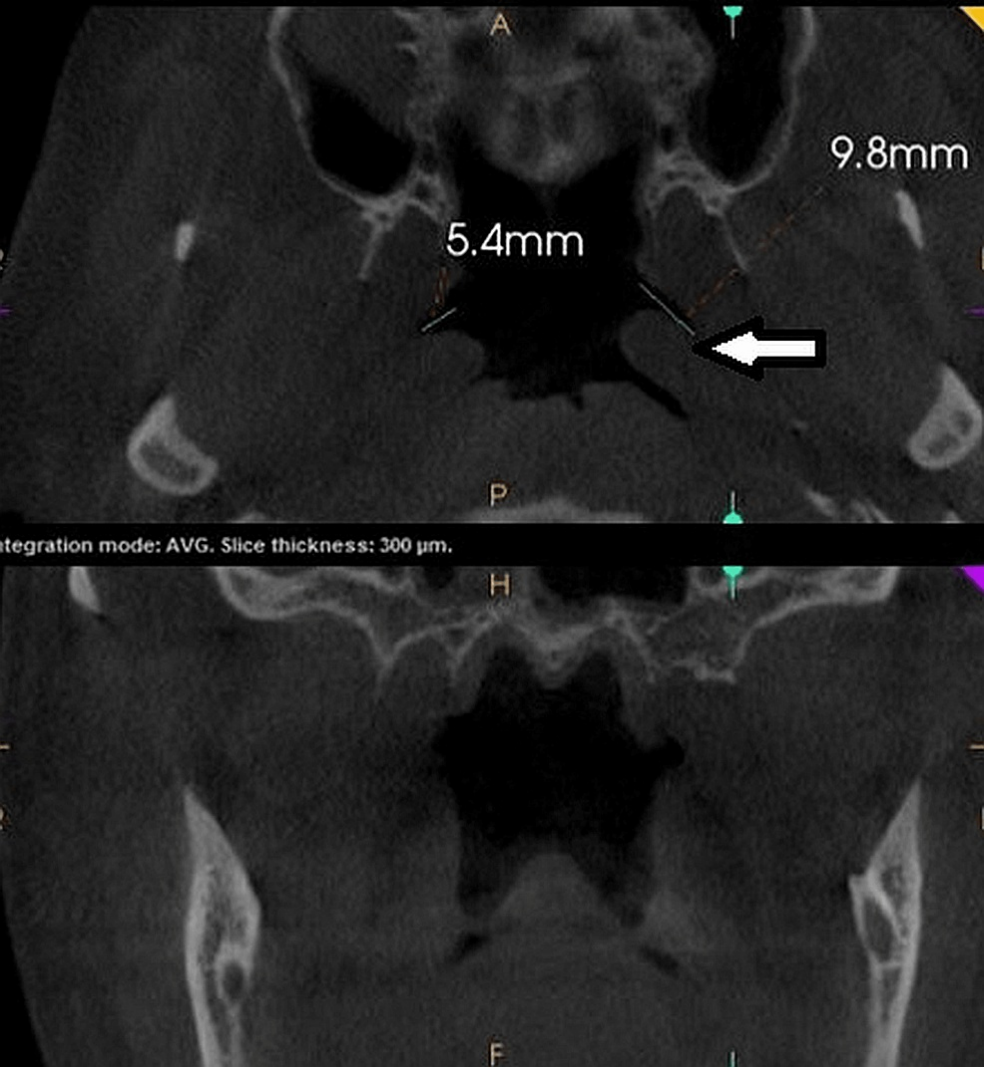
CBCT PNS view to evaluate the eustachian tube

Procedure for CBCT: The patient was posted for CBCT (CS-9300, Care Stream Dental CBCT unit, Voxel size 180 μm, Kv 90, mA6.3, sec 8; version 3.7.1.0 (31/8/2016) analysis. CBCT - PNS view was taken for each individual patient in a standing position. Eustachian tube length and volume were calculated using the axial sections at the level of the root tips of maxillary first molars (Figure 4).

**Figure 5:**
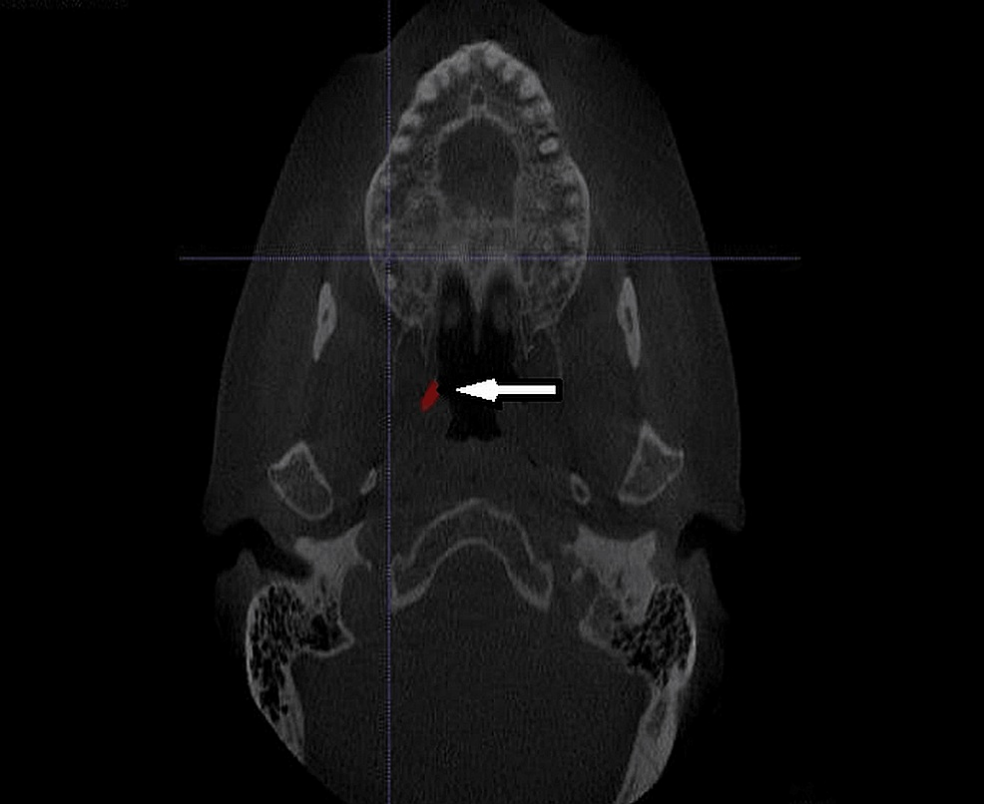
Volumes of ET was measured on software

Initially, the image acquisition was done. X-rays were generated, and the multiple images of 1x1mm windows were captured by the detector. The field of view was set to 16 X 15. The multiplanar or 3D images obtained were reconstructed for further assessment. In the oblique sections, the horizontal or axial plane was adjusted to the root tip of the upper first molar. Then the length of ET was calculated by using measuring tools, i.e., from the opening of the eustachian tube to the maximum radiolucent part of the canal in that section. The volumes of ET were also measured for both the left and right ear using third-party software (ITK - SNAP, 3.6 version, April-1-2017). The CBCT images of normal, healthy individuals, as a control, were procured from an existing database of individuals who were age and sex-matched, and ET values of length and volume were measured (Figure 5).

Once the values were obtained, they were entered into an Excel sheet (MS Word) (Redmond, USA) and subjected to statistical analysis using the SPSS Inc. Released 2009. PASW Statistics for Windows, Version 18.0. Chicago: SPSS Inc. A p-value of ≤0.05 was considered statistically significant.

## Results

Prevalence of oral submucous fibrosis in two genders, i.e., males and females, in the present study. There were a total of 40 OSMF patients, out of whom 34 (85%) are males and six (15%) are females.

The age distribution of the study population in OSMF instances showed that six patients were over the age of 51, 10 patients were between the ages of 31 and 40, 19 patients were between the ages of 41 and 50, and five patients were under the age of 30. The study's participants' ages ranged from 17 to 80 years old.

Table 1 shows the comparison of the volume of ET (mm3) of OSMF patients with different functional grades (M1, M2, M3, and M4) with left and right ears by Kruskal-Wallis ANOVA analysis.

**Table 1:** Comparison of OSMF functional grades (M1, M2, M3, and M4) with left and right ear ET-volume (mm3) by Kruskal-Wallis ANOVA OSMF: Oral submucous fibrosis, ET: Eustachian tube, CBCT: Cone-beam computed tomography

Grades	Left ear CBCT-volume			Right ear CBCT-volume		
	Mean	SD	Mean rank	Mean	SD	Mean rank
Grade M1	8.73	4.73	28.13	8.68	3.18	31.00
Grade M2	4.77	2.47	18.13	5.11	2.08	18.33
Grade M3	4.64	2.46	18.60	5.23	2.82	18.13
Grade M4	5.16	0.19	22.00	4.27	0.69	12.50
H-value	4.4480			8.5220		
P-value	0.2170			0.0360		
Pair wise comparisons by Mann-Whitney U test						
M1 vs M2	p=0.0528			p=0.0081		
M1 vs M3	p=0.0707			p=0.0169		
M1 vs M4	p=0.4334			p=0.1172		
M2 vs M3	p=0.9504			p=0.9174		
M2 vs M4	p=0.5510			p=0.3711		
M3 vs M4	p=0.7656			p=0.5510		

This reveals there is a significant difference in volume for the right ear (p= 0.036), but there is no significance in the left ear. On pairwise comparisons by the Mann-Whitney U test, i.e., the intra-group comparisons, there was a significant difference in the volume of ET when comparing M1 and M2 (p= 0.008) and M1 and M3 (p= 0.016). The p-value obtained was not significant when comparing M1 and M4; M2 and M3; M2 and M4; and M3 and M4 on the right side, and there was no significant difference on the left side.

Table 2 shows the correlations among all parameters in the right and left ear in the OSMF group.

**Table 2:** Correlations among all parameters of the right and left ears in the OSMF group OSMF: Oral submucous fibrosis, CBCT: Cone-beam computed tomography

Variables	Sides		CBCT-		CBCT-			
			volume (mm3)		length (mm)		Audiometry	
			Left ear	Right ear	Left ear	Right ear	Left ear	Right ear
CBCT-volume (mm3)	Left ear	Correlation	-					
									p-value	-					
									Right ear	Correlation	0.781	-				
		p-value	<0.001	-												
	CBCT-length (mm)	Left ear	Correlation	0.612	0.571	-											
			p-value	<0.001	<0.001	-				
									Right ear		Correlation	0.522	0.57	0.882	-		
		p-value	<0.01	<0.001	0<0.001	-											
Audiometry										Left ear	Correlation	-0.342	-0.372	-0.348	-0.382	-	
		p-value	0.0310	0.0180	0.0280	0.0150	-										
									Right ear		Correlation	-0.274	-0.162	-0.074	-0.206	0.272	-
	p-value	0.088	0.317	0.652	0.203	0.089	-										

There is a positive correlation when comparing the CBCT volumes and lengths of ET in the right and left ears. However, there was a negative correlation obtained when comparing the audiometry of the right and left ear with CBCT lengths and CBCT volumes. The outcomes were significant when it came to the right ear's CBCT volume, the left ear's CBCT length, and the left ear's audiometric correlations. In a relationship between audiometry and the right ear, the p-value was not significant.

## Discussion

The prevalence of OSMF in the Indian subcontinent is about 0.2% to 6.3% [10], has increased from 0.03% to 6.42% in the last four decades, and is predominantly seen in the middle age group. A few studies have shown the occurrence of the disease in the younger and older age groups as well. Both genders are affected, but a higher proportion of men are affected than women, with a ratio ranging from 2.7:1 to 6.8:1 [10,11]. The advanced stages of OSMF have a malignant transformation rate varying from 7%-13%, and in long-term studies, it is about 7.6%, as reported by Jani YV et al. [12].

Multiple etiological factors have been proposed, with areca nut consumption being the most important causative factor. Other factors include the usage of other tobacco products like gutka, the consumption of spicy food, nutritional deficiencies, etc. The atrophic changes within the mucosa can lead to a marble-like appearance of the mucosa, along with blanching, depigmentation, the formation of vesicles, and ulcerations. On palpation, there is reduced elasticity of the oral mucosal tissues along with leathery mucosa and the presence of palpable fibrous bands in the retromolar region and around the oral cavity [6].

Studies have revealed that patients with OSMF have partial hearing loss, which has been documented by the use of audiometry in such patients [13,14]. Imaging of the ET was always done by traditional imaging methodologies like computed tomography (CT) or magnetic resonance imaging (MRI). The ET is composed of both bone and soft tissue. The CT is usually used to visualize the bony components, and the MRI is for the soft tissue components. However, with the advent of CBCT, both the bony and soft tissue components could be visualized with fewer radiation doses than with traditional CT. Hence, the alterations or changes in the ET length and volume were visualized using the CBCT in this study. As per the current study, based on the gender prevalence, it was observed that the majority were male patients compared to females. The ratio is 5.6:1, which is in accordance with the studies of Chawla H et al. [6] and Kumar KK et al. [15]. The age of occurrence of OSMF has a very wide range, with cases reported as young as eight years [16] in children to 80 years in individuals. However, the average age of incidence of OSMF is 31 to 40 years. In this study, the highest number of cases was seen in the age group of 41 to 50 years. Hence, this study varies with the normal age distribution, with cases seen in a higher age range than normal.

All the patients who were graded both clinically and functionally were subjected to an audiometric evaluation. The audiometry was conducted for both ears, independently for the right ear and the left ear. The audiometric values in various clinical grades in this study did not show any significance for both ears. The pairwise comparisons within the group also did not show any significance in all the groups. This could be because the fibrotic changes in various grades may not be so severe, leading to changes in the audiometric values. Even the S4 grading did not reveal any differences in the audiometric values because S4 is only given when a possibly malignant lesion is present, even though the functional grade is M1. Hence, a purely different classification in regard to the palatal changes in OSMF may be helpful to assess and explain the changes in the audiometric values more accurately. Hence, this classification falls short of addressing the issues of audiometric changes in OSMF [10].

The audiometric values were also compared as per their functional grading, and the values in various grades also did not show any significance in both ears. The pairwise intergroup comparisons were not significant. The functional grading is mainly based on the mouth opening of the individual, which is reduced due to the formation of fibrotic bands around the oral cavity and the retromolar region. The fibrotic bands and the amount of fibrosis in the palate involving the muscles of the soft palate, which can lead to the loss of patency of the ET, were not considered as part of the functional grading. Hence, the changes in the ET are mainly attributed to the fibrosis of the palatal muscles, so the audiometric changes are independent of the functional grading [17-20].

For the assessment of the correlation between the audiometric values obtained in OSMF patients and the length and volume by CBCT, it was found that there was a negative correlation between the audiometric values and the CBCT length and volume of ET. Most of the cases of OSMF showed mild to moderate hearing loss in both ears; however, this loss was not very significant with the increase in grades of OSMF. However, the length and volume of the ET was decreasing with the increase in the grades of OSMF. Hence, it is difficult to authenticate the hearing loss with the reduction in the length and volume of the ET measured on CBCT alone.

The study's limitations include the use of only cases with oral submucous fibrosis and the absence of controls for the audiometric analyses. Instead of considering the entire length of the eustachian tube, which includes both the bone and soft tissue components, just the cartilaginous portion is taken into account. Additional investigations using a laryngoscope could have been considered to do the clinical assessment of the patency of the Eustachian tube [21], which was a drawback as it was not performed. Furthermore, the smaller number of patients or subjects taken in each group for this study is also a limitation of this study. The cost of the CBCT image evaluation was also a factor in enrolling fewer subjects in the study. Limitations in viewing the soft tissue component of the eustachian tube in the CBCT are that the measurements of the eustachian tube in the CBCT were done only in axial planes, so the extent of the soft tissue component cannot be assessed exactly in our CBCT study. This poses limitations when seeing the soft tissue component of the eustachian tube. The Valsalva maneuver could not be performed on our patients in our study during the imaging of the eustachian tube due to fibrosis and stiffness of the buccal mucosa. No previously identified publications or studies describing the use of imaging in the identification and management of eustachian tube disorders were available. Because of the complicated eustachian tube's structure, location, orientation, and mixed tissue composition, no imaging method has consistently been shown to be better overall. As a result, this study had its limits in properly predicting the actual outcome of hearing loss in OSMF patients. Comparing the hearing loss in OSMF patients with the changes in the length and volume of the ET led to the hypothesis that, while the hearing loss is observed due to an increase in OSMF grade, it cannot be proven that these patients' conductive deafness is also caused by anatomical variations in the size and volume of their ET. Better imaging techniques may therefore be needed to quantify ET in OSMF instances in order to forecast the actual morphological alterations brought on by hearing loss in these patients.

## Conclusions

Therefore, from the present study, it can be concluded that all OSMF cases should be evaluated for hearing deficit, and imaging of the eustachian tube should be part of the OSMF assessment for morphological changes of the ET that may cause hearing deficit. Although cone-beam computed tomography provides the current best combination of spatial resolution and position, further studies can be taken up in this area.

## References

[1] Scgwartz J (2022). CiNii: Atrophia idiopathica mucosa oris. 11th International Dental Congress.

[2] Ariyawardana A, Athukorala AD, Arulanandam A (2006). Effect of betel chewing, tobacco smoking and alcohol consumption on oral submucous fibrosis: a case-control study in Sri Lanka. J Oral Pathol Med.

[3] Hebbar PB, Sheshaprasad R, Gurudath S, Pai A, Sujatha D (2014). Oral submucous fibrosis in India: Are we progressing??. Indian J Cancer.

[4] Pindborg JJ, Sirsat SM (1966). Oral submucous fibrosis. Oral Surg Oral Med Oral Pathol.

[5] Tilakaratne WM, Klinikowski MF, Saku T, Peters TJ, Warnakulasuriya S (2006). Oral submucous fibrosis: review on aetiology and pathogenesis. Oral Oncol.

[6] Chawla H, Urs AB, Augustine J, Kumar P (2015). Characterization of muscle alteration in oral submucous fibrosis-seeking new evidence. Med Oral Patol Oral Cir Bucal.

[7] Smith ME, Scoffings DJ, Tysome JR (2016). Imaging of the Eustachian tube and its function: a systematic review. Neuroradiology.

[8] Gupta SC, Singh M, Khanna S, Jain S (2004). Oral submucous fibrosis with its possible effect on eustachian tube functions: A tympanometric study. Indian J Otolaryngol Head Neck Surg.

[9] More CB, Gupta S, Joshi J, Varma SN (2012). Classification system for oral submucous fibrosis. J Indian Acad Oral Med Radiol.

[10] Auluck A, Hislop G, Poh C, Zhang L, Rosin MP (2009). Areca nut and betel quid chewing among south asian immigrants to western countries and its implications for oral cancer screening. Rural Remote Health.

[11] Shah N, Sharma PP (1998). Role of chewing and smoking habits in the etiology of oral submucous fibrosis (OSF): a case-control study. J Oral Pathol Med.

[12] Jani Jani, Yesha V, Dudhia Dudhia, Bhavin B (2016). The clinicohistopathologic study of oral submucous fibrosis: A new staging system with treatment stragies. J Indian Acad Oral Med Radiol.

[13] Badra S, Fathima Fathima, Mahesh Mahesh (2015). Evaluation of hearing efficiency in patients with oral sub mucous fibrosis. J Pharm Sci Res.

[14] Sowbhagya M, Shivhare P, Yadav M (2016). Audiometric and tympanometric assessment in patients with oral submucous fibrosis. BJMMR.

[15] Kiran Kumar K, Saraswathi TR, Ranganathan K, Uma Devi M, Elizabeth J (2007). Oral submucous fibrosis: a clinico-histopathological study in Chennai. Indian J Dent Res.

[16] Mundra RK, Gupta SK, Gupta Y (1999). Oral submucous fibrosis in paediatric age group. Indian J Otolaryngol Head Neck Surg.

[17] Sudo M, Sando I, Suzuki C (1998). Three-dimensional reconstruction and measurement study of human eustachian tube structures: a hypothesis of eustachian tube function. Ann Otol Rhinol Laryngol.

[18] Bluestone CD, Bluestone MB (2005). Eustachian Tube Structure, Function, Role in Otitis Media. Hamilton: B C Decker.

[19] Leuwer R, Schubert R, Kucinski T, Liebig T, Maier H (2002). The muscular compliance of the auditory tube: a model-based survey. Laryngoscope.

[20] Cohn AM (1977). Clinical assessment of eustachian tube ventilatory function. Laryngoscope.

[21] Todd NW (2000). There are no accurate tests for eustachian tube function. Arch Otolaryngol Head Neck Surg.

